# Capturing heat illness in vulnerable populations through the lens of older adults: a scoping review protocol of health administrative data

**DOI:** 10.1136/bmjopen-2025-111726

**Published:** 2026-05-26

**Authors:** Justine-Gaëlle St-Arnaud, Caroline Raymond, Soumaïla Boukari Abdou, Ferdaous Roussafi, Isabelle Dufour, François M Castonguay

**Affiliations:** 1 Department of Health Management, Evaluation and Policy, University of Montreal School of Public Health, Montreal, Quebec, Canada; 2 Centre for Public Health Research (CReSP), University of Montreal, Montreal, Quebec, Canada; 3 Observatoire québécois des inégalités, Montreal, Quebec, Canada; 4 School of Nursing, Faculty of Medicine and Health Sciences, Université de Sherbrooke, Sherbrooke, Quebec, Canada

**Keywords:** Climate Change, Health informatics, PUBLIC HEALTH, GERIATRIC MEDICINE

## Abstract

**Abstract:**

**Introduction:**

Heatwaves are among the fastest-growing climate-related threats to human health, increasing in frequency, intensity and duration with climate change. Older adults are disproportionately affected, reflecting intersecting physiological, social and economic vulnerabilities. Beyond mortality, heatwaves drive substantial but often under-recognised morbidity, including emergency visits and hospitalisations for cardiovascular, respiratory, renal and metabolic conditions. Health administrative data provide valuable opportunities to improve understanding of these phenomena and for the quantification of their impacts. However, comparability is limited by heterogeneity in case definitions, that is, the criteria used to determine which health events are counted as cases: some studies only clinically diagnosed heat illness, while others also capture outcomes plausibly triggered or exacerbated by heat. It is further constrained by differences in International Classification of Diseases (ICD) versions, national adaptations, coding practices and adoption timelines across countries. No synthesis has yet mapped these heterogeneous approaches for older adults, despite their over-representation among those most affected by heat-health risks. This heterogeneity limits the ability to capture the true burden of disease and to inform adaptation planning.

**Methods and analysis:**

We will conduct a scoping review to map how heat-related diagnoses among older adults are identified and measured in health administrative data. The review will follow the initial methodological framework of Arksey and O’Malley refined by Levac *et al* and adhere to the PRISMA-ScR (Preferred Reporting Items for Systematic Reviews and Meta-Analyses extension for Scoping Reviews) guidelines. The search strategy will be developed with a public health librarian and applied to MEDLINE (Ovid), Embase and Web of Science. We will include peer-reviewed and grey literature published in English or French from 2010 onward. Two reviewers will independently screen titles, abstracts and full texts in Covidence, with disagreements resolved by consensus or a third reviewer. Data will be extracted using a standardised form to capture study characteristics, ICD codes, definitions of heat exposure and approaches to measurement. A descriptive and thematic analysis will be conducted, and findings will be presented narratively and in tables.

**Ethics and dissemination:**

Ethical approval is not required for this review as it involves secondary analysis of published and publicly available data; for more information, contact University of Montreal’s Research Integrity department at *plaintes-crr@umontreal.ca*. Results will be published through a peer-reviewed publication, conference presentations and knowledge transfer activities with public health stakeholders in Québec. This review contributes to the MEDICCS (*Modélisation économique des impacts des changements climatiques sur la santé*) project and will support recommendations for improving the capture of heat-related morbidity in health administrative data.

**STRENGTHS AND LIMITATIONS OF THIS STUDY**

- Targets a sentinel population (older adults) and systematically maps International Classification of Disease (ICD) coding practices, case definitions and measurement approaches in health administrative data, enhancing policy and surveillance relevance.
- Applies established scoping review methodology (Arksey & O’Malley, Levac *et al*) and PRISMA-ScR (Preferred Reporting Items for Systematic Reviews and Meta-Analyses extension for Scoping Reviews) guidelines, ensuring rigour, transparency and reproducibility.
- Heterogeneity in coding practices, case definitions and exposure metrics across studies may limit comparability, although results will be reported transparently to maximise interpretability.

## Introduction

 In the face of climate change, heatwaves, defined as a sustained period of temperatures exceeding usual climatic conditions of a region, are increasingly recognised as one of the fastest-growing climate-related hazards to human health.^1^ Research shows that heatwaves are rising not only in frequency but also in intensity and duration, thereby placing an enormous burden on health and social systems.^2^ With increasing global warming, high-temperature events are expected to become more frequent and intense, whereas low-temperature events are projected to decline consistently at both global and continental scales, across nearly all inhabited regions.^1^ However, definitions of heatwaves are heterogeneous across the literature, with no widely agreed-upon definition, which is reflected in the variation of quantitative approaches, including the heat metric used, minimum duration and intensity threshold, depending on geographic and contextual factors.^3^ In Québec, for example, research often uses 90th/95th percentile daily mean threshold over ≥2 days, whereas the official definition relies on fixed maximum and minimum temperature thresholds over ≥3 days.^4^

Evidence shows that global exposure to extreme heat has increased substantially, especially among older adults, as a result of both population ageing and climate change.^2^ Projections indicate that by mid-century, the proportion of persons aged 69 years or older living in regions where the 95th percentile of daily maximum temperature exceeds 37.5°C is expected to rise from 14% to over 23%.^5^ Heat-related mortality among older adults is also rising, driven by population ageing, which amplifies the overall mortality burden, and by increased vulnerability due to physiological changes, higher prevalence of chronic disease and reduced ability to adapt to heat.^6 7^ A recent analysis of 854 cities across 30 European countries over 20 years estimated that heat accounts for approximately 20 000 deaths annually, representing 0.69% of all deaths, with 40% of the total mortality burden occurring among the oldest age group (85+).^8^

Beyond mortality, heatwaves trigger a much larger and often hidden burden of morbidity, including emergency visits and hospitalisations for heat-related illnesses, as well as exacerbations of all-cause conditions such as acute renal failure, cardiovascular and respiratory diseases, diabetes, electrolyte imbalance, mental health disorders, nephritis and more.^9 10^ Because these chronic conditions have multiple aetiologies, the contribution of heat is often overlooked in clinical coding, resulting in systematic underestimation of its true burden.^11^ Health administrative data, covering nearly the entire population and providing longitudinal follow-up, are widely used to quantify the health impacts of heat and evaluate adaptation measures, making them powerful tools for assessing the impacts of extreme heat.^9 12 13^ Variability in coding practices, measurement tools, populations and clinical presentation results in highly heterogeneous approaches to identifying and measuring heat-related diagnoses.^13^ Standardisation efforts are not yet universally implemented, limiting comparability across studies and settings.

These challenges are further compounded by the evolution of the International Classification of Diseases (ICD), the global standard for health information developed by the WHO, which has undergone several revisions (ICD-9, ICD-10, ICD-11). Countries have adopted these versions at different times, making comparisons of heat-related morbidity data across locations and periods difficult.^14^,^16^ This temporal and geographic heterogeneity complicates the comparability of case definitions, coding practices and morbidity data collection.^17^ To date, no synthesis has mapped these diverse case definitions specifically for older adults. This group represents a pragmatic sentinel population for wider vulnerability, embodying the intersection of physiological, social and economic risks.^6 18 19^ Biological ageing (impaired thermoregulation, multimorbidity, medication effects), combined with social and economic constraints (isolation, mobility limits, lower cooling access), concentrates risk. Equity considerations are therefore central, as the health impacts of extreme heat are shaped by social determinants of health and distributed unevenly within and across populations. Applying an equity lens highlights how intersecting disadvantages influence both vulnerability and the ways heat-related morbidity is identified and measured. Heatwaves thus generate strong, early signals in this population, useful for surveillance. Given rapid population ageing and projected increases in dangerous heat exposure among older adults globally, prioritising this sentinel group also advances equity and preparedness.

This scoping review is part of a broader research project (Projet MEDICCS *—Modélisation économique des impacts des changements climatiques sur la santé*), which aims to estimate the economic costs of heatwaves and extreme heat in the Canadian province of Québec and to evaluate the benefits of adaptation measures. This project seeks to inform the development of a provincial heat-health adaptation plan that benefits the Québec government, municipalities and vulnerable populations.^20^ While the primary focus is on Québec, our review casts a broader net. By examining the international scientific literature on how heat-related morbidity is identified in health administrative data during heatwaves, we will highlight transferable algorithms and validation practices and then adapt the best evidence to Québec’s context to strengthen recommendations for policymakers.

This scoping review will therefore:

1. Examine how heat-related conditions in older adults are captured in health administrative datasets during documented periods of elevated ambient heat exposure (including heatwaves and extreme heat time series), by identifying the ICD codes used.
2. Identify and summarise existing evidence on the validity and reliability with which these datasets capture emergency department visits, hospitalisations and other healthcare encounters occurring during heatwaves periods or triggered by extreme heat.

Answering these questions is essential for generating accurate burden-of-disease estimates and for assessing the effectiveness of heat-health action plans. Preliminary validation studies suggest substantial under-ascertainment and geographic variability in coding practices, underscoring the need for a systematic synthesis.^15 21^

## Methods and analysis

### Protocol design

The review started in July 2025 and is expected to be completed by July 2026. The protocol for this scoping review was registered in the Open Science Framework.^22^ This study will follow the five-stage methodological framework developed by Arksey and O’Malley^23^ and refined by Levac *et al*^24^: (1) identifying the research question; (2) identifying relevant studies; (3) selecting studies; (4) collecting data; and (5) collating, summarising and reporting outcomes. The review will also adhere to the PRISMA-ScR (Preferred Reporting Items for Systematic Reviews and Meta-Analyses extension for Scoping Reviews) guidelines.^25^

#### Stage 1: identification of the research question

Following extensive consultation with the research team and a preliminary review of the existing literature, the central question guiding this scoping review was formulated as: How are heat-related diagnoses among older adults identified and measured in health administrative data? Older adults were prioritised as a proxy for populations presenting with vulnerability factors when examining diagnostic patterns in the context of extreme heat, reflecting both the depth of existing research and their disproportionate burden of heat-related illness driven by intersecting vulnerabilities. A subset of questions has been further created to hone the focus of the review and guide the extraction and analysis of relevant data:

1. What medical diagnoses are most frequently associated with heatwaves among older adults?
2. Which International Classification of Disease (ICD) codes are used to designate these diagnoses?
3. How are heat-sensitive illnesses measured and reported in health administrative data?

#### Stage 2: identification of relevant studies

The research strategy (table 1) was developed in collaboration with the public health-related librarian at the Université de Montréal and has followed an iterative process, beginning with a pilot search in MEDLINE (Ovid) to refine the final search approach. For example, the MEDLINE (Ovid) strategy is:

(‘Aged’ OR ‘Frailty’ OR ‘Geriatrics’ OR ‘Aging’ OR ‘Elder’ OR ‘Senior’ OR ‘Gerontologic’ OR ‘Older adult’ OR ‘Older patient’ OR ‘Older citizen’ OR ‘Older stakeholder’ OR ‘Older individual’ OR ‘Older population’ OR ‘Old person’ OR ‘Old people’ OR ‘Older men’ OR ‘Older women’ OR ‘Aging population’) AND (‘High temperature’ OR ‘Hot temperature’ OR ‘Elevated temperature’ OR ‘Above average temperature’ OR ‘Extreme ambient temperature’ OR ‘High ambient temperature’ OR ‘Hot spell’ OR ‘Hot weather’ OR ‘Severe weather’ OR ‘Thermal exposure’ OR ‘Climate extreme’ OR ‘Extreme hot weather’ OR ‘Heat stress disorder’ OR ‘Heat exhaustion’ OR ‘Heat stroke’ OR ‘Extreme heat’ OR ‘Intense heat’ OR ‘Heat stress’ OR ‘Heat index’ OR ‘Climate crisis’ OR ‘Global warming’ OR ‘Greenhouse effect’ OR ‘Climate change’ OR ‘Severe heat’ OR ‘Prolonged heat’ OR ‘Heat spell’ OR ‘Heat exposure’ OR ‘Unusual heat’ OR ‘Persistent heat’ OR ‘Record breaking heat’ OR ‘Acute heat’ OR ‘Chronic heat’ OR ‘Environmental heat’ OR ‘Heatwave’ OR ‘Thermal exposure’ OR ‘Heat anomaly’) AND (‘Emergency medical services’ OR ‘Health effect’ OR ‘Health impact’ OR ‘Disease burden’ OR ‘Adverse health effect’ OR ‘Heat-related illness’ OR ‘Heat-related morbidity’ OR ‘Heat-related disease’ OR ‘Heat-related anxiety’ OR ‘Peripheral neuropathy’ OR ‘Digestive system disease’ OR ‘Respiratory tract disease’ OR ‘Urogenital abnormalities’ OR ‘Cardiovascular diseases’ OR ‘Nutritional and metabolic diseases’ OR ‘Morbidity’ OR ‘Mortality’ OR ‘Stroke’ OR ‘Suicide’ OR ‘Epilepsy’ OR ‘Migraine disorders’ OR ‘Headache’ OR ‘Cognitive dysfunction’ OR ‘Mental disorder’ OR ‘Gout’ OR ‘Chronic disease’ OR ‘COPD’).

**Table 1:** Main keywords per concepts for the search strategy

Main concept	Keywords	
Older adults	Ageing population Elder Elderly Senior Gerontologic Geriatrics	Older adult /stakeholder Older patient /citizen Older individual /population Old person /people Older men /women Frailty
Extreme heat	High /hot temperature Elevated /above average temperature Extreme /high ambient temperature Hot spell/weather Thermal exposure Severe weather Climate extreme Extreme hot weather Heat stress disorder Heat exhaustion Heat stroke Extreme/intense heat Heat stress/index	Climate crisis Global warming Greenhouse effect Climate change Extreme /intense heat Heat stress /index Severe /prolonged heat Heat spell /exposure Unusual /persistent heat Record breaking heat Acute /chronic heat Environmental heat Heat anomaly/wave Thermal exposure
Morbidity and diagnosis	Hospital admissions Medical consultations Diagnosis code T67/X30/E86/ICD Hospitalisation Health services Emergency medical services Suicide Epilepsy Migraine disorders Headache Cognitive dysfunction Mental disorder Gout Chronic obstructive pulmonary disease (COPD)	Health impact /effect Disease burden Adverse health effect Heat-related illness /morbidity Heat-related disease /anxiety Peripheral neuropathy Digestive system disease Respiratory tract disease Urogenital abnormalities Cardiovascular diseases Nutritional and metabolic diseases Morbidity /mortality Stroke Chronic disease

ICD, International Classification of Diseases.

Additional databases include Embase and Web of Science. The full search strategy is available in the online supplemental file. The selection of health outcomes and diseases was guided by preliminary evidence from the literature on conditions shown to be exacerbated by extreme heat. In addition to general categories frequently used in heat-health research (eg, cardiovascular, respiratory, renal, etc), we included specific conditions which have been identified in prior studies as potentially heat-sensitive.^13 26^ This approach ensures comprehensive coverage of both well-established and emerging heat-related health outcomes. The search will retain English and French articles published after 2010 and will include both published and unpublished (grey) literature.

Consistent with the Johanna Briggs Institute recommendations for scoping reviews,^27^ the *Population, Concept and Context* (PCC) framework was applied to define the key concepts of the research question and to guide the search strategy and inclusion criteria. In this review, *Population* refers to the older adults, used as a proxy for the broader vulnerable population. *Concept* corresponds to the primary subject of the investigation—morbidity and diagnosis in this group. *Context* represents the setting of interest, which is exposure to extreme heat. A set of keywords corresponding to the three main concepts was developed to construct the search equations and will be applied to titles and abstracts. Search results will be exported to Covidence via the Université de Montréal to facilitate screening. Duplicates will be removed using this platform, and the selected articles will be imported into EndNote.

The inclusion criteria are as follows: original quantitative and qualitative research, as well as grey literature, and articles addressing the main concepts: extreme heat, older adults, morbidity and their corresponding diagnoses, published in English or French from 2010 onward. Grey literature will be identified through targeted searches of government health agencies (such as Health Canada or Centers for Disease Control and Prevention), international organisation (eg, WHO), institutional repositories, surveillance guidelines and technical documents not indexed in peer-reviewed databases. Furthermore, studies explicitly reporting ICD codes or describing case definitions or equivalent approaches related to health administrative data, will be included to ensure capture of relevant evidence.

Letters to editors, editorials, perspectives, conference proceedings, opinion pieces, posters and interviews will be excluded. We will also not consider unavailable full text, those published in other languages and literature published before 2010. Articles published prior to 2010 were excluded to ensure relevance to contemporary coding practices and diagnostic standards. Earlier studies are less comparable due to substantial changes in ICD revisions, national adoption timelines, and evolving approaches to defining and measuring heat-related morbidity.^13^,^16^

#### Stage 3: study selection

Two reviewers (J-GS-A and SBA) will independently screen articles against the eligibility criteria, first by examining titles and abstracts, and second by reading full texts. Any disagreement between the two reviewers will be resolved through discussion, with consultation of a third reviewer (CR or FMC) if needed. A pilot test will be performed on the first 50 titles and abstracts to identify sources of disagreement and refine eligibility criteria. An algorithm will be developed to support the reviewers, and a flowchart generated from Covidence will document the screening process.

#### Stage 4: data charting

##### Data extraction

A standardised form will be developed and iteratively refined by the two reviewers to capture the relevant data. Following Levac *et al*,^24^ to ensure the reliability of the extraction process, both reviewers will independently extract the data from the first 5–10 articles to assess consistency and identify any necessary revisions. Full data extraction will then be conducted in parallel by the two reviewers. The following data will be extracted:

- Article metadata.
  - Title.
  - Author(s).
  - Year of publication.
  - Objectives.
  - Study design.
- Data source.
  - Countries where the research was conducted.
  - Population/sample size.
  - Inclusion and exclusion criteria.
  - Name/type database used.
  - Geographic and year coverage.
- Heat exposure.
  - Heat event source.
  - Exposure definition of heat.
  - Metric.
  - Data linkage method.
- Heat-related health outcomes.
  - Diagnostic codes or classifications/health administrative data.
  - Specific codes used.
  - Case definition.
- Measurement/operationalisation.
  - Decision rules applied (algorithm).
  - Unit of measurement (hospitalisation, emergency visit, death certificate, etc).
  - Statistical approach.
  - Comparative/reference period.
- Equity considerations.
  - How are they addressed.
- Priorities and gaps in research.
  - Limitations.
  - Recommendations.

Reference tables (tables2  3) linking ICD codes with heat-related metrics with their corresponding versions will facilitate consistency in data extraction among reviewers and comparison across studies. This scoping review uses ICD-10 (rather than ICD-9 or ICD-11) because it is the most widely adopted internationally. Accordingly, we include studies from 2010 onward to minimise transition-related (from ICD-9 to ICD-10) heterogeneity and reduce miscoding linked to coding changes.

**Table 2:** International Classification of Disease 10th version (ICD-10) linking heat-related metrics

Code^28^	ICD-10 title	Definition (incl.)
T67	Effects of heat and light	Disorders due to heat and light, eg, heatstroke/sunstroke (T67.0); heat syncope/collapse (T67.1); heat cramp (T67.2); heat exhaustion, anhydrotic— heat prostration due to water depletion (T67.3); heat exhaustion due to salt depletion (T67.4); heat exhaustion, unspecified (T67.5); heat fatigue, transient (T67.6); heat oedema (T67.7); other effects of heat and light (T67.8); unspecified (T67.9)
E86	Volume depletion	Dehydration Depletion of volume of plasma or extracellular fluid Hypovolaemia
X30	Exposure to excessive natural heat (external cause)	Excessive natural heat as the cause of sunstroke Exposure to heat, not otherwise specified (NOS)

**Table 3:** Code equivalence for heat-related morbidity

ICD-10 Code and title	ICD-9 Equivalence^29^	ICD-11 Equivalence^30^
T67 Effects of heat and light	992 Effects of heat and light	NF01 Effects of heat
E86 Volume depletion	276.5 Volume depletion	5C70 Volume depletion
X30 Exposure to excessive natural heat (external cause)	E900 Excessive heat	PB15 Unintentional exposure to excessive heat

ICD, International Classification of Diseases.

### Stage 5: collating, summarising and reporting outcomes

#### Data analysis

We will conduct a descriptive and thematic analysis of the extracted data.^24^ The descriptive analysis will summarise the characteristics of the included studies, while the thematic analysis will identify and categorise approaches to the identification and measurement of heat-related diagnoses using health administrative data. Particular attention will be given to: (1) how heat-related health outcomes are defined and coded; (2) the methodological approaches used to capture and measure heat exposure; (3) equity considerations; and (4) data limitations or gaps. Findings will be presented in tables and a narrative summary, highlighting patterns, divergence, research gaps and implications for future studies.

### Patient and public involvement

Patients and the public did not participate in, nor were involved in, the development of this protocol.

## Ethics and dissemination

This scoping review relies solely on the collection and analysis of data from published and publicly available sources, and consequently, does not require research ethics approval. No human participants will be directly involved, and no identifiable personal or sensitive data will be collected. No risks are expected to arise from conducting this review.

The review will generate a structured synthesis of how heat-related morbidity among older adults is defined and captured in health administrative data. Through an analysis of ICD coding practices, case definitions and measurement approaches, it will identify sources of heterogeneity that currently limit comparability, undermine validity across studies and utility of administrative data for capturing heat-related morbidity. By focusing on older adults as a sentinel population, it will shed light on broader vulnerabilities to extreme heat and the challenges of accurately estimating its true burden. The evidence assembled will serve as a reference point for strengthening the validity and consistency of surveillance methods in future research and practice.

Dissemination will occur through multiple pathways. Findings will be submitted for publication in peer-reviewed journals in public health, epidemiology and climate-health research, and presented at provincial, national and international scientific conferences. The outputs of this review will also directly contribute to the *Modélisation économique des impacts des changements climatiques sur la santé* (MEDICCS) project. In addition to scholarly dissemination, tailored knowledge-translation activities will be undertaken with public health stakeholders in Quebec. These efforts aim to ensure that the synthesised evidence informs adaptation planning, surveillance strategies and policies designed to better protect older adults from the health risks of extreme heat.

### Strength and limitations

This scoping review has several strengths. Its targeted focus on older adults as a sentinel group, who show early and pronounced heat-related signals, enhances the policy relevance of the findings. By mapping how heat-related morbidity is captured in health administrative data, the review increases the utility of findings for surveillance and adaptation planning. By examining key elements, such as case definitions, ICD coding practices, data sources and heat-exposure metrics, it provides a structured overview that highlights both enablers and barriers to comparability. We apply transparent, reproducible scoping methods, including comprehensive database searches, multiple stages of screening and data extraction, and procedures consistent with PRISMA-ScR guidelines.

Nevertheless, several limitations must be acknowledged. Publication bias may arise if inconclusive findings are less likely to be disseminated. Variations across studies, particularly in case definitions, coding practices and exposure metrics, will restrict cross-study comparability. Language and temporal boundaries may exclude some relevant work, and administrative data can under-ascertain heat’s contribution. To mitigate these issues, we will group studies by common methodological features and transparently present the diversity in the evidence to draw meaningful conclusions within these constraints.

## Supplementary material

10.1136/bmjopen-2025-111726 online supplemental file 1
