# Supplementary material for: Capturing heat illness in vulnerable populations through the lens of older adults: a scoping review protocol of health administrative data
Source: BMJ Open. 2026 May 26;16(5):e111726. doi: 10.1136/bmjopen-2025-111726 (PMC13218177; doi:10.1136/bmjopen-2025-111726)
Supplement: online supplemental file 1 [file bmjopen-16-5-s001.pdf]

Supplemental file – Adapted search strategies per database

Database	Search strategy
MEDLINE (Ovid)	1 exp Global Warming/<br>2 exp Greenhouse Effect/<br>3 Climate Change/<br>4 exp Extreme Heat/<br>5 exp Hot Temperature/<br>6 exp Heat Stress Disorders/ or exp Heat Exhaustion/ or exp Heat Stroke/<br>7 ((heat* adj2 (stress* or intense or severe or index or prolonged or spell or unusual or "record breaking" or persistent or exposure or acute or chronic or environmental or anomal* or wave*)) or (temperature* adj2 (high or "above average" or elevated or "high ambient" or extreme*)) or (hot adj2 (spell* or weather*)) or (thermal adj2 exposure) or (severe adj2 weather) or (climate adj2 extreme*) or "extreme hot weather" or "climate crisis" or ("heat-related" adj2 (illness* or morbidit* or disease*)) or "heat anx*").ab,kf,ti.<br>8 1 or 2 or 3 or 4 or 5 or 6 or 7<br>9 exp Aged/<br>10 exp Frailty/<br>11 exp Geriatrics/<br>12 (ag?ing or elder* or senior* or geronto* or (old* adj2 (adult* or patient* or citizen* or stakeholder* or individual* or population* or person* or people or m?n or wom?n)) or "aging population*").ab,kf,ti.<br>13 9 or 10 or 11 or 12<br>14 exp digestive system diseases/ or exp respiratory tract diseases/ or exp urogenital abnormalities/ or exp cardiovascular diseases/ or exp "skin and connective tissue diseases"/ or exp "nutritional and metabolic diseases"/ or exp endocrine system diseases/<br>15 exp Morbidity/<br>16 exp Mortality/<br>17 exp Hospitalization/<br>18 exp Health Services/<br>19 exp emergency medical services/ or emergency room visits/<br>20 "International Classification of Diseases"/<br>21 Stroke/

	22 Venous Thromboembolism/<br>23 Pulmonary Disease, Chronic Obstructive/<br>24 exp Chronic Disease/<br>25 Gout/<br>26 exp Mental Disorders/<br>27 Cognitive Dysfunction/<br>28 Headache/<br>29 Migraine Disorders/<br>30 Epilepsy/<br>31 Suicide/<br>32 ((hospital adj2 admission*) or (medical adj2 consultation*) or (health adj2 (impact* or effect*)) or "disease burden" or "adverse health effect*" or "peripheral neuropath*" or "diagnosis code*" or T67* or X30* or E86*).ab,kf,kw,ti.<br>33 14 or 15 or 16 or 17 or 18 or 19 or 20 or 21 or 22 or 23 or 24 or 25 or 26 or 27 or 28 or 29 or 30 or 31 or 32<br>34 8 and 13 and 33
EMBASE	1 exp greenhouse effect/<br>2 climate change/<br>3 exp extreme hot weather/<br>4 exp high temperature/<br>5 exp heat injury/ or exp heat exhaustion/ or exp heat stroke/<br>6 ((heat* adj2 (extreme* or stress* or intense or severe or index or prolonged or spell or unusual or "record breaking" or persistent or exposure or acute or chronic or environmental or anomal* or wave*)) or (temperature* adj2 (high or "above average" or elevated or "high ambient" or extreme*)) or (hot adj2 (spell* or weather*)) or (thermal adj2 exposure) or (severe adj2 weather) or (climate adj2 extreme*) or "extreme hot weather" or "climate crisis" or ("heat-related" adj2 (illness* or morbidit* or disease*)) or "heat anx*").ab,kf,kw,ti.<br>7 1 or 2 or 3 or 4 or 5 or 6<br>8 exp aged/<br>9 exp frailty/<br>10 exp geriatrics/<br>11 (ag$ing or elder* or senior* or geronto* or (old* adj2 (adult* or patient* or citizen* or stakeholder* or individual* or population* or person* or people or m?n or wom?n)) or "aging population*").ab,kf,kw,ti.<br>12 8 or 9 or 10 or 11<br>13 exp digestive system disease/<br>14 exp respiratory tract disease/<br>15 exp urogenital tract malformation/<br>16 exp cardiovascular disease/

	17 exp skin disease/<br>18 exp connective tissue disease/<br>19 exp metabolic disorder/<br>20 exp endocrine disease/<br>21 exp morbidity/<br>22 exp mortality/<br>23 exp hospitalization/<br>24 exp health service/<br>25 exp emergency health service/<br>26 exp emergency department visit/<br>27 "International Classification of Diseases"/<br>28 cerebrovascular accident/<br>29 venous thromboembolism/<br>30 lung disease/<br>31 exp chronic disease/<br>32 gout/<br>33 exp mental disease/<br>34 cognitive defect/<br>35 headache/<br>36 migraine/<br>37 epilepsy/<br>38 suicide/<br>39 ((hospital adj2 admission*) or (medical adj2 consultation*) or (health adj2 (impact* or effect*)) or "disease burden" or "adverse health effect*" or "peripheral neuropath*" or "diagnosis code*" or T67* or X30* or E86*).ab,kf,kw,ti.<br>40 13 or 14 or 15 or 16 or 17 or 18 or 19 or 20 or 21 or 22 or 23 or 24 or 25 or 26 or 27 or 28 or 29 or 30 or 31 or 32 or 33 or 34 or 35 or 36 or 37 or 38 or 39<br>41 7 and 12 and 40
Web of Science	TS=((global warming) OR (heat* NEAR/2 (extreme* OR stress* OR intense OR severe OR index OR prolonged OR spell OR unusual OR "record breaking" OR persistent OR exposure OR acute OR chronic OR environmental OR anormal* OR wave* OR stroke OR exhaustion OR anx*)) OR (temperature* NEAR/2 (high OR "above average" OR elevated OR "high ambient" OR extreme* OR hot)) OR (hot NEAR/2 (spell* OR weather*)) OR (thermal NEAR/2 exposure) OR (severe NEAR/2 weather) OR (climate NEAR/2 (extreme* OR change* OR crisis)) OR (greenhouse effect*) OR ("extreme hot weather") OR ("heat-related" NEAR/2 (illness* OR mobidit* OR disease*))) AND TS=((frailty) OR (geriatric*) OR (elder*) OR (senior*) OR (geronto*) OR ((old* OR ag$ing OR aged) NEAR/2 (adult* OR patient* OR citizen* OR stakeholder* OR individual* OR population* OR person* OR people OR m?n OR wom?n))) AND TS=("urogenital abnormalit*" OR "skin and connective tissue disease*" OR "nutritional and

	metabolic disease" OR "endocrine system disease*" OR morbidity OR hospitalization* OR (emergency NEAR/2 ("medical service*" OR "room visit*")) OR "International Classification of Diseases" OR stroke OR (venous NEAR/2 thromboembolism) OR COPD OR gout OR (cognitive NEAR/2 dysfunction*) OR headache OR epilepsy OR suicide OR (hospital NEAR/2 admission*) OR (medical NEAR/2 consultation*) OR "disease burden" OR "adverse health effect*" OR (peripheral NEAR/2 neuropath*) OR (diagnosis NEAR/2 code) OR T67* OR X30* OR E86* OR ICD9 OR ICD10 OR (disease* NEAR/2 ("digestive system" OR "respiratory tract" OR cardiovascular OR chronic)) OR (disorder* NEAR/2 (migraine OR mental)) OR (health NEAR/2 (impact* OR effect* OR service*)) OR (imbalance* NEAR/2 (water OR electrolyte*)) OR dehydration)
