# Supplementary material for: Capturing heat illness in vulnerable populations through the lens of older adults: a scoping review protocol of health administrative data
Source: BMJ Open. 2026 May 26;16(5):e111726. doi: 10.1136/bmjopen-2025-111726 (PMC13218177; doi:10.1136/bmjopen-2025-111726)
Supplement: Supplementary file 2 [file bmjopen-2025-111726.reviewer_comments.pdf]

PEER REVIEW HISTORY

BMJ Open publishes all reviews undertaken for accepted manuscripts. Reviewers are asked to complete a checklist review form and are provided with free text boxes to elaborate on their assessment. These free text comments are reproduced below.

ARTICLE DETAILS

Title (Provisional)

Capturing Heat Illness in Vulnerable Populations Through the Lens of Older Adults: A Scoping Review Protocol of Health Administrative Data

Authors

St-Arnaud, Justine-Gaëlle; Raymond, Caroline; Boukari Abdou, Soumaïla; Roussafi, Ferdaous; Dufour, Isabelle; Castonguay, François M.

VERSION 1 - REVIEW

**Reviewer**	**1**
**Name**	**Chaseling, Georgia**
**Affiliation**	**The University of Sydney**
**Date**	**12-Jan-2026**
**COI**	**None**

The authors present their protocol for a scoping review assessing health administration data related to morbidity and heatwaves. I have no major concerns with this manuscript but have a few point below that should be addressed.

1. The title does not adequatley reflect this work. I would suggest the authors include the word(s) 'a protocol' somewhere in the title to ensure a reader is aware this is not the results of the scoping review.

2. The primary aim of this study is to "Examine how heat-related conditions in older adults are captured in health administrative datasets during documented heatwave periods", however, I feel as though the search terms and inclusion/exclusion criterea do not allow of this specific analysis. This could be as simple as noting within the inclusion criterea that manuscripts must document a specific heatwave period only, and exclude the general time series analysis looking at the effect of hot temperatures on morbidity. These are two separate ideas - and so if the authors are only interested in specific heatwaves events, this must be reflected within the methodology.

3.The other aim mentions "Evaluate whether these datasets reliably capture emergency department visits, hospitalisations, and other healthcare encounters occurring during heatwaves

periods" however the methodology does not provide information how this evaluation will occur. How do the authors plan on evaluating the reliability of the data capture?

4. The inclusion of commentaries seems contradicting considering the exclusion of perspective and opinion pieces. could the authors please comment on why commentaries would be included?

5. What does 'reviewing validation studies' mean within the aims.

VERSION 1 - AUTHOR RESPONSE

Comments to the Author from Reviewer 1

The authors present their protocol for a scoping review assessing health administration data related to morbidity and heatwaves. I have no major concerns with this manuscript but have a few points below that should be addressed.

1. The title does not adequately reflect this work. I would suggest the authors include the word(s) 'a protocol' somewhere in the title to ensure a reader is aware this is not the results of the scoping review.

- **Response:** Thank you very much for this clarifying point. We have adjusted the title by adding the word "protocol" to reflect the nature of this manuscript.

- **Line 1-2:** "Capturing Heat Illness in Vulnerable Populations Through the Lens of Older Adults: A Scoping Review Protocol of Health Administrative Data"

2. The primary aim of this study is to "Examine how heat-related conditions in older adults are captured in health administrative datasets during documented heatwave periods", however, I feel as though the search terms and inclusion/exclusion criterea do not allow of this specific analysis. This could be as simple as noting within the inclusion criterea that manuscripts must document a specific heatwave period only, and exclude the general time series analysis looking at the effect of hot temperatures on morbidity. These are two separate ideas - and so if the authors are only interested in specific heatwaves events, this must be reflected within the methodology.

- **Response:** We thank the reviewer for raising this ambiguity. We believe that restricting the inclusion criteria to documented heatwave periods only would exclude a substantial portion of the literature where extreme heat is assessed via continuous temperature metrics or threshold-based definitions without being tied to a single, named event. We also believe that it would reduce generalizability,

  because the definition of "heatwave" varies substantially across jurisdictions and studies whereas general time series often capture locally relevant extremes. To improve alignment, we have revised the "documented heatwave periods" for

"periods of elevated ambient heat exposure (including heatwaves and extreme heat time series)" in our primary aim.

- **Line 166 – 16G:** "Examine how heat-related conditions in older adults are captured in health administrative datasets during documented periods of elevated ambient heat exposure (including heatwaves and extreme heat time series), by identifying the ICD codes used.

3. The other aim mentions "Evaluate whether these datasets reliably capture emergency department visits, hospitalisations, and other healthcare encounters occurring during heatwaves periods" however the methodology does not provide information how this evaluation will occur. How do the authors plan on evaluating the reliability of the data capture?

- **Response:** We agree that the wording of this aim implied a formal evaluation or reliability, while the methods do not specify such an approach. The intend of this scoping review is to map and summarise existing evidence on data capture reliability relevant to healthcare encounters during extreme heat periods. The scoping review itself will not generate new reliability estimates or conduct an original reliability exercise. Rather, its purpose is to identify what reliability evidence exists, what measures have been used, and importantly what gaps remain to inform the subsequent empirical phase of the broader research project funded by the grant. It is in the latter that we will judge dataset fitness. Accordingly, we have revised the aim to replace "evaluate" with wording more consistent with a scoping review, i.e., "identifying and summarizing existing evidence".

- **Line 170 – 173:** "Identify and summarize existing evidence on the validity and reliability with which these datasets capture emergency department visits, hospitalisations, and other healthcare encounters occurring during heatwaves periods or triggered by extreme heat."

4. The inclusion of commentaries seems contradicting considering the exclusion of perspective and opinion pieces. could the authors please comment on why commentaries would be included?

- **Response:** We thank the reviewer for highlighting this inconsistency. We agree that including "commentaries" was not aligned with our exclusion of perspectives and opinions (Line 246). We have therefore revised our inclusion criteria and removed "commentaries in the peer-reviewed journals".

- **Previously at Line 238.**

5. What does 'reviewing validation studies' mean within the aims.

- **Response:** We agree that the phrase "Reviewing available validation studies" was not sufficiently clear within the aims and could be interpreted as implying that the review would conduct a formal validation assessment. To avoid any ambiguity and to keep the aim aligned with the descriptive, mapping purpose of a scoping review, we have removed this phrase.

- **Line 166 – 16G: "**Examine how heat-related conditions in older adults are captured in health administrative datasets during documented periods of elevated ambient heat exposure (including heatwaves and extreme heat time series), by identifying the ICD codes used."
